# P-606. Pneumococcal Serotype Distribution Among People Living With HIV (PLWH) in the German CAPNETZ-Cohort

**DOI:** 10.1093/ofid/ofaf695.819

**Published:** 2026-01-11

**Authors:** Jacob Gerstenberg, Grit Barten-Neiner, Florian Voit, Norbert Suttorp, Christoph Boesecke, Christian Hoffmann, Daiana Stolz, Mathias W Pletz, Gernot Rohde, Martin Witzenrath, Marcus Panning, Andreas Essig, Jan Rupp, Olaf Degen, Christoph Stephan, Benjamin T Schleenvoigt

**Affiliations:** Institute of Tropical Medicine, Tübingen University Hospital / Eberhard-Karls University, Tübingen, Germany, Tübingen, Baden-Wurttemberg, Germany; CAPNETZ STIFTUNG, Hannover, Germany, Hannover, Niedersachsen, Germany; TUM University Hospital Rechts der Isar Ismaninger Str. 22 81675 Munich, Germany, Munich, Bayern, Germany; Department of Infectious Diseases and Respiratory Medicine, Charité-Universitätsmedizin Berlin, Corporate Member of Freie Universität Berlin and Humboldt-Universität zu Berlin, Germany, Berlin, Berlin, Germany; Department of Internal Medicine I, University Hospital Bonn, Bonn, Germany, Bonn, Nordrhein-Westfalen, Germany; ICH Study Center Hamburg, Hamburg, Germany, Hamburg, Hamburg, Germany; Department of Pneumology, University Medical Center Freiburg, Freiburg, Germany, Freiburg, Baden-Wurttemberg, Germany; Institute of Infectious Diseases and Infection Control, Jena University Hospital/Friedrich-Schiller-University, Jena, Germany, Jena, Thuringen, Germany; University Hospital Frankfurt, Frankfurt am Main, Hessen, Germany; Charité-Universitätsmedizin Berlin, Berlin, Brandenburg, Germany; Institute of Virology, University Medical Center-University of Freiburg, Freiburg, Germany, Freiburg, Baden-Wurttemberg, Germany; Institute of Medical Microbiology and Hygiene, University Hospital of Ulm, Ulm, Germany, Ulm, Baden-Wurttemberg, Germany; Department of Infectious Diseases and Microbiology, University Hospital Schleswig-Holstein, Lübeck, Germany, Lübeck, Schleswig-Holstein, Germany; University Hospital Hamburg Eppendorf, Hamburg, Germany, Hamburg, Hamburg, Germany; Medical Department II, Section Infectious Diseases, University Medical Center, Frankfurt am Main, Germany, Frankfurt am Main, Hessen, Germany; Institute of Infectious Diseases and Infection Control, Jena University Hospital/Friedrich-Schiller-University, Jena, Germany, Jena, Thuringen, Germany

## Abstract

**Background:**

Community-acquired pneumonia (CAP) is a major cause of hospitalization among people living with HIV (PLWH), with *Streptococcus pneumoniae* identified as the leading pathogen. Identifying pneumococcal serotypes is critical for optimizing vaccination strategies, as different vaccines target distinct serotypes. While pneumococcal conjugate vaccines (PCVs) and polysaccharide vaccines (PPSVs) have been widely used, their serotype coverage has not been investigated within the subpopulation of PLWH. This study investigated the distribution of pneumococcal serotypes among PLWH in the German CAPNETZ cohort and compared it with an age- and sex-matched HIV-negative control group.

**Figure 1:**
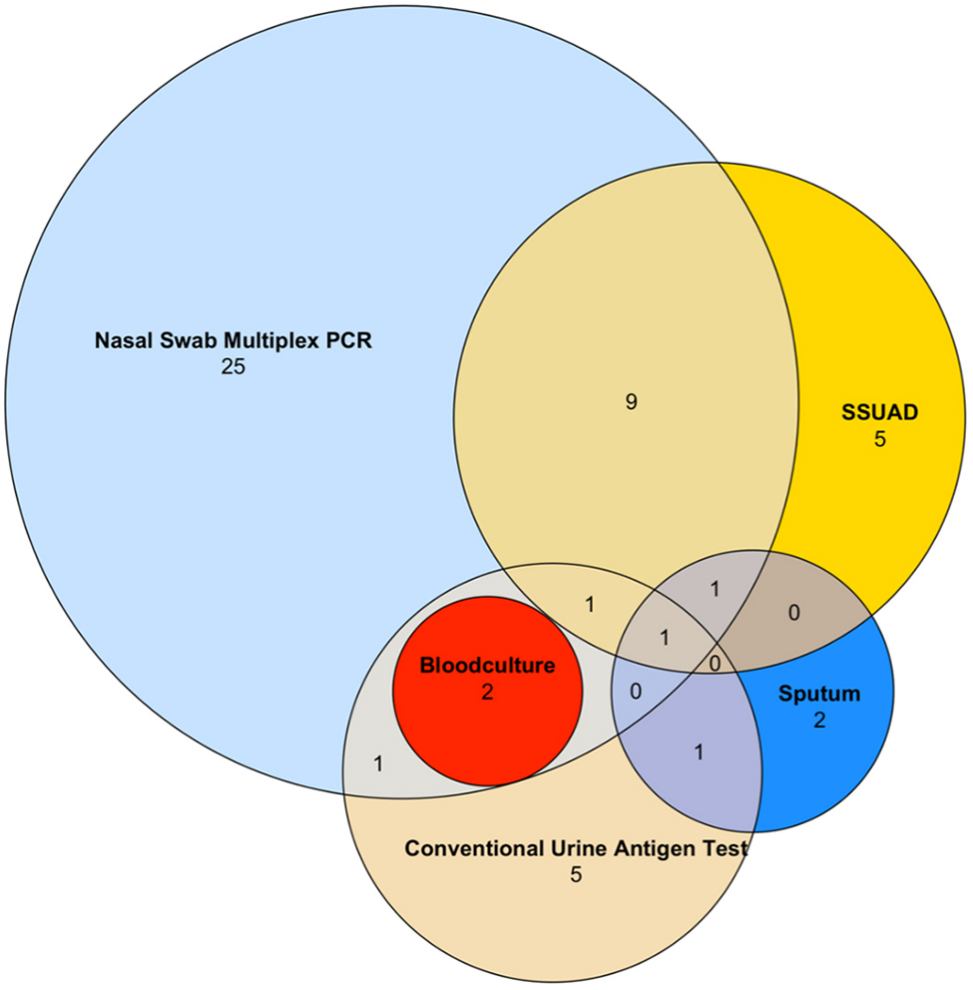
Comparison of different microbiological testing for participants with S. pneumoniae community acquired pneumoniae. A total of n=56 participants were tested positive for S. pneumoniae on any of the above listed method. However, the graphic only illustrates n=53 cases, as complete depiction of multiple overlaps is not feasible. The three missing cases were all positive for blood cultures.

**Table 1:**
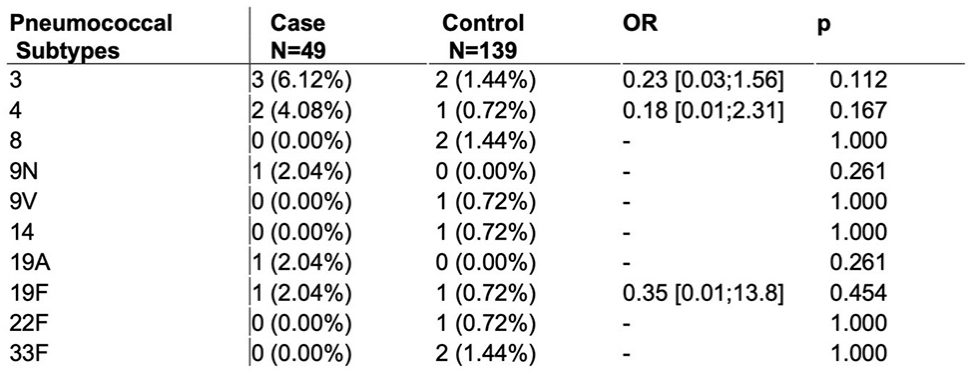
Distribution of S. pneumoniae serotypes among case and control group based on serotype-specific urine antigen detection assays.

**Methods:**

A total of 73 HIV-positive participants with CAP were matched in a 1:3 ratio with 218 HIV-negative controls. Pneumococcal detection was conducted using microbiological tests, including blood cultures, sputum analysis, nasopharyngeal swabs, conventional pneumococcal urine antigen tests (PUAT), with additional serotype-specific urine antigen detection (SSUAD) assays carried out at Pfizer’s vaccine research center (NY).

**Table 2:**
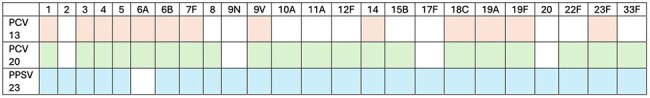
Vaccine coverage on S. pneumoniae serotypes of common commercially available pneumococcal vaccines.

**Results:**

Of the 291 participants, 55 were positive for *Streptococcus pneumoniae*, mostly identified by nasal swab PCR (n=40) followed by SSUAD (n=19). From these, the most identified serotype was serotype 3, followed by serotype 4. No significant differences in serotype distribution were observed between the case and control group. All serotypes detected in the case group were covered by PPSV23, but not by PCV13 or PCV20, although it was only one case outside this coverage.

**Conclusion:**

Distribution of pneumococcal serotypes among PLWH with CAP in the CAPNETZ cohort was similar to that of HIV-negative individuals. The study findings confirm that the vast majority of detected serotypes could be covered by both PPSV23 as well as PCV 20. More studies with larger sample size are needed, to confirm these preliminary data.

**Disclosures:**

Florian Voit, MD, B Braun Melsungen AG: Grant/Research Support|Gilead Sciences: Honoraria|Janssen-Cilag: Travel support|MSD: Grant/Research Support|Pfizer: Travel support|ViiV Healthcare: Travel support Mathias W. Pletz, MD, GSK: Advisor/Consultant|GSK: Honoraria|MSD: Advisor/Consultant|MSD: Honoraria|Pfizer: Advisor/Consultant|Pfizer: Grant/Research Support|Pfizer: Honoraria|Shionogi: Advisor/Consultant|Shionogi: Honoraria Christoph Stephan, MD, AbbVie: Advisor/Consultant|AbbVie: Expert Testimony|Gilead Sciences: Advisor/Consultant|Gilead Sciences: Expert Testimony|Janssen-Cilag: Advisor/Consultant|Janssen-Cilag: Expert Testimony|Merck: Advisor/Consultant|Merck: Expert Testimony|Shionogi: Advisor/Consultant|Shionogi: Expert Testimony|ViiV Healthcare: Advisor/Consultant|ViiV Healthcare: Expert Testimony Benjamin T. Schleenvoigt, MD, AIDS-Hilfe Dresden: Advisor/Consultant|AIDS-Hilfe Potsdam: Advisor/Consultant|AstraZeneca: Advisor/Consultant|DAH: Advisor/Consultant|Falk: Advisor/Consultant|Gilead: Advisor/Consultant|Gilead: Grant/Research Support|Gilead: Congress support|IQWiG: Advisor/Consultant|Janssen: Advisor/Consultant|Mitteldeutsche HIV-Behandler e.V.: Grant/Research Support|MSD: Advisor/Consultant|MSD: Congress support|Pfizer: Advisor/Consultant|ViiV: Advisor/Consultant|ViiV: Grant/Research Support

